# Phytochrome F mediates red light responsiveness additively with phytochromes B1 and B2 in tomato

**DOI:** 10.1093/plphys/kiad028

**Published:** 2023-01-21

**Authors:** Daniel Balderrama, Samantha Barnwell, Keisha D Carlson, Elsa Salido, Ruby Guevara, Christina Nguyen, Andreas Madlung

**Affiliations:** Department of Biology, University of Puget Sound, Tacoma, WA 98416, USA

## Abstract

Phytochromes are red light and far-red light sensitive, plant-specific light receptors that allow plants to orient themselves in space and time. Tomato (*Solanum lycopersicum*) contains a small family of five phytochrome genes, for which to date stable knockout mutants are only available for three of them. Using CRISPR technology, we created multiple alleles of *SlPHYTOCHROME F* (*phyF*) mutants to determine the function of this understudied phytochrome. We report that SlphyF acts as a red/far-red light reversible low fluence sensor, likely through the formation of heterodimers with SlphyB1 and SlphyB2. During photomorphogenesis, phyF functions additively with phyB1 and phyB2. Our data further suggest that phyB2 requires the presence of either phyB1 or phyF during seedling de-etiolation in red light, probably via heterodimerization, while phyB1 homodimers are required and sufficient to suppress hypocotyl elongation in red light. During the end-of-day far-red response, phyF works additively with phyB1 and phyB2. In addition, phyF plays a redundant role with phyB1 in photoperiod detection and acts additively with phyA in root patterning. Taken together, our results demonstrate various roles for SlphyF during seedling establishment, sometimes acting additively, other times acting redundantly with the other phytochromes in tomato.

## Teaser

CRISPR-induced knockout mutants suggest additive roles in red light responses for phytochrome F with phytochromes A, B1, and B2 in tomato.

## Introduction

As permanently sessile organisms it is important for plants to optimally orient themselves in space and over developmental time to gain access to life-sustaining resources, including light and water. Roots have to grow into the ground to provide the plant with water and soil-borne nutrients and to anchor the plant firmly in the ground. Stems help to position leaves to attain an optimal balance between maximal exposure to sunlight and the need to conserve water. Flowers and fruit have to be positioned accessibly for pollinators or seed dispersers, and finally all these requirements have to be achieved in a way that provides the most favorable three-dimensional shape for the plant to guarantee its structural stability and metabolic efficiency (Teichmann and Muhr, 2015).

Plants have evolved mechanisms to sense and respond to environmental cues from germination to maturity to develop the best possible body architecture. One mechanism with which plants shape their bodies is achieved by sensing light quality and quantity with photoreceptors and translating the information to guide specific growth patterns (Kami et al., 2010; Chen and Chory, 2011; Xu et al., 2015). Information gathered via photoreceptors is relayed via a multitude of signal transduction pathways that lead to developmental and physiological responses (Leivar and Quail, 2011; Sheerin and Hiltbrunner, 2017). Light perception and its signal transduction intersect with many other components of plant development, such as the sensing of temperature (Kumar et al., 2012; Jung et al., 2016; Legris et al., 2019), water status (Boccalandro et al., 2009), metabolic pathways, osmoregulation, and the accumulation of nutrients (Moran, 2007). One important aspect of integration and long-distance signal transduction of multiple signals is via the phytohormones, which can link the perception of the environment with the developmental response (Lau and Deng, 2010).

Phytochromes are chromoproteins that consist of a light-absorbing chromophore and an apoprotein, which transmits the light signal to downstream transducers (Li et al., 2015). Light is received via a chromophore that is covalently bound to the apoprotein, which changes conformation upon irradiation with either red light (R) or far-red light (FR) (Rockwell et al., 2006). Phytochrome's chromophore generally activates the protein upon reception of R and deactivates it after absorption of FR, although different members of the phytochrome gene family have different activation requirements.

Although phytochromes have been found in many species, it is interesting that DNA sequences of this gene family are often not well conserved between species. In Arabidopsis (*Arabidopsis thaliana*), five *PHYTOCHROME* (*PHY*) genes have been characterized: *PHYA, PHYB, PHYC, PHYD,* and *PHYE* (Clack et al., 1994; Mathews and Sharrock, 1997). Functions of *PHYs* overlap somewhat but each *PHY* also plays distinct roles in many developmentally and environmentally controlled plant responses (Whitelam et al., 1993; Halliday et al., 1994; Tepperman et al., 2006; Franklin et al., 2007; Sheehan et al., 2007; Franklin and Quail, 2010; Kiyota et al., 2010). Phytochromes act as dimers (Sharrock and Clack, 2004), forming both homo- or heterodimers that can elicit different responses depending on the type of combination of proteins in the dimer (Sánchez-Lamas et al., 2016). An Arabidopsis mutant devoid of all functional *PHY* genes was shown to have very severe deficiencies in germination and development, especially in R conditions, and was only marginally fertile in the delayed flowering time mutant background *flowering locus T* (*ft*) (Strasser et al., 2010). Functions for each phytochrome are by no means exclusive and interactions of several phytochromes in the same physiological response are common (Weller et al., 2001; Tepperman et al., 2004).

Phytochromes have been studied most intensely in Arabidopsis, using genetic, biochemical, and molecular approaches. Phylogenetic analysis has shown that phyA and phyB are ubiquitously found in all seed plants (Casal et al., 2014), while occurrence and phylogenetic origin of the other phytochromes vary between species (Mathews, 2010). Like Arabidopsis, tomato (*Solanum lycopersicum*) contains a five-member *PHY* gene family (Alba et al., 2000). Those five genes encoding the phytochrome apoproteins are named *PHYA*, *PHYB1*, *PHYB2*, *PHYE*, and *PHYF*. In Arabidopsis the *PHY* genes each belong to phylogenetically divergent groups, comprising amino acid identities generally between each other of 69%–76%, although *AtPHYD* and *AtPHYB* display greater sequence identity (90%) (Clack et al., 1994; Pratt et al., 1995). In Arabidopsis, *PHY* divergence likely occurred via three major gene duplication events. The first separated *PHYA/C* from the other *PHYs*. The second separated *PHYA* from *PHYC,* and *PHYB/D* from *PHYE*. The third occurred after divergence of the Brassicaceae and separated *PHYB/D* into separate *PHYB* and *PHYD* genes (Smith, 2000). Phytochromes in tomato (Solanaceae) have not undergone the same phylogenetic evolution. Tomato has two genes that are similar to *AtPHYB* (*SlPHYB1* and *SlPHYB2*). These two genes arose by a gene duplication event affecting the Solanaceae after their divergence from the Brassicaceae (Pratt et al., 1995) explaining why *AtPHYB* and *SlPHYB1* show differences in function (Lazarova et al., 1998). In contrast to Arabidopsis, mutation of *SlphyB1* in tomato results only in temporarily red light insensitivity at a very young seedling stage (the mutant was therefore originally named *tri*), and as adults these mutants look very similar in phenotype to WT tomato (Lazarova et al., 1998). In contrast to *AtphyB, SlphyB1* mutants respond to FR (van Tuinen et al., 1995b), underscoring the notion that tomato and Arabidopsis *PHYB* genes are functionally different.

*SlPHYB2* appears to play a role in early seedling development (Hauser et al., 1998), and in cooperation with *SlPHYA* and *SlPHYB1,* in the control of de-etiolation (Weller et al., 2000). Our recently published work (Carlson et al., 2020) shows that *SlPHYB1* and *SlPHYB2* have both unique and redundant functions, for example in the regulation of photosynthetic activity (antagonistic), gravitropic and phototropic (unique) responses, and adventitious root formation (redundant). Like Arabidopsis, tomato has a *PHYA* gene that mediates responses to FR (van Tuinen et al., 1995a; Shichijo et al., 2001). Arabidopsis mutants for *PHYA* do not have a pronounced phenotype in white light, suggesting a lesser importance of *PHYA* in the plant's sensing of broad spectrum light (Whitelam et al., 1993). Adult tomato plants with a mutation in *PHYA* (far-red light insensitive, originally named *fri)* display a smaller, slightly wilted-looking phenotype (van Tuinen et al., 1995a). Interestingly, SlphyA also reduces the elongation of shoot length of seedlings grown in the dark, possibly by using phyA activated in the embryo while still on the mother plant (Carlson et al., 2019). Taken together, *SlPHYA*, *SlPHYB1*, and *SlPHYB2* have distinct roles for some responses, while showing genetic redundancy in others (Kendrick et al., 1994, 1997; Quail, 1997; Smith and Whitelam, 1997; Weller et al., 2000; Carlson et al., 2020).

Aside from these three *PHY* genes there are two additional *PHY* genes in tomato: *SlPHYE* and *SlPHYF*. *SlPHYF* is the most likely ortholog of *AtPHYC*, (Alba et al., 2000), but its physiological function in tomato is largely unknown. *SlPHYE* of tomato is phylogenetically similar to *AtPHYE* (Hauser et al., 1995; Pratt et al., 1997). A study using an artificial microRNA approach to down-regulate the gene suggested a role for *SlPHYE* in the shade avoidance response (Schrager-Lavelle et al., 2016). While phyA and phyB are the obligatory phytochromes found in all species, phyC, phyD, and phyE appear to function mostly in cooperation with phyB (at least in Arabidopsis), fine-tuning and extending the functions of phyB through heterodimerization (Mathews, 2010).

Mutants in three tomato phytochrome genes (*SlPHYA*, *SlPHYB1*, *SlPHYB2*) are available (van Tuinen et al., 1995a; van Tuinen et al., 1995b; Lazarova et al., 1998) from the mutant collection of the Tomato Genetics Resource Center (TGRC, http://tgrc.ucdavis.edu/) but knockout mutants for tomato *SlPHYE* and *SlPHYF* have not been available. Functions for these genes have been proposed based on transcriptional levels of these genes in various tissues of tomato. *SlPHYE* and *SlPHYF* have the least abundant transcripts among the five PHY family members of tomato, both in dark- and light-grown tissue of young seedlings less than ten days old (Hauser et al., 1998). While *SlPHYE* abundance increases dramatically in older tissues, *SlPHYF* expression spikes in about 10 d old seedlings and then levels out at slightly lower than peak levels throughout the plant's adult life (Hauser et al., 1998).

Here we report the construction and phenotypic analysis of stable, CRISPR-induced knockout alleles in *SlPHYF*. Our results suggest that PHYF is involved in de-etiolation, the photoperiod and end-of-day FR responses, as well as in root patterning.

## Results

### Construction and verification of CRISPR mutants

**Figure 1:**
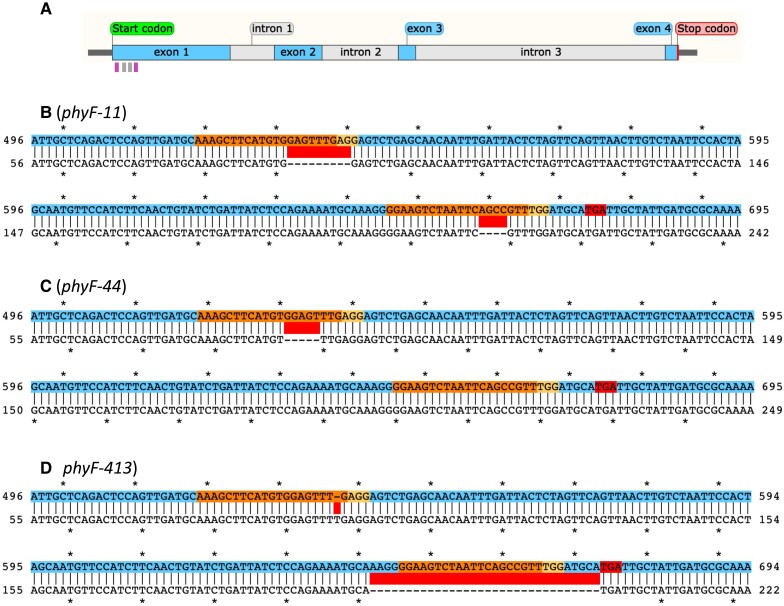
Molecular structure of CRISPR-induced *phyF* alleles. A: Structure of *PHYF* indicating the location of gRNA target sequences in exon 1 and the location of the genotyping primers (see Supplemental Table 1). The forward and reverse priming sites are indicated by the left and right pink vertical bars, respectively, underneath the gene model. The gRNA target sequences are shown as gray vertical bars under the gene model. B-D: Alignments of mutations in *phyF*-11, *phyF*-44, and *phyF*-413, respectively. The color-coded sequence (top line) depicts part of exon 1 of wild-type *PHYF*. The gRNA target sites and the corresponding protospacer adjacent motif (PAM) sites are shown in orange and yellow, respectively. Insertion and deletion mutations are indicated by red bars between the sequences. Premature TGA stop codons resulting from CRISPR-induced mutations are indicated in the top sequence in red.

We used CRISPR technologies to generate *phyF* knockout mutants. We isolated three CRISPR *phyF* alleles (*phyF-11*, *phyF-44*, and *phyF-413*), which contained independent knockout mutations, and which had lost the Cas9 transgene during selfing and segregation of homozygous mutants. Sanger sequencing showed that the *phyF-11* allele has a 9 bp deletion in its 5′ gRNA target sequence and a 4 bp deletion in its 3′ gRNA target sequence leading to an early STOP codon 11 bp downstream of the mutation (Figure 1). The *phyF-44* allele has a single 5 bp deletion in the 5′ gRNA target region leading to an early STOP 173 bp after the mutation (Figure 1). Finally, the *phyF-413* allele is characterized by a 1 bp insertion in the 5′ gRNA target sequence and an early STOP immediately following a 32 bp deletion in the 3′ gRNA target region (Figure 1). Given that all three CRISPR alleles are nonsense mutations resulting in the loss of more than 75% of the protein's sequence, including the suspected chromophore attachment region, dimerization domain, and the nuclear localization domain, it seems highly likely that the mutations in all three alleles result in a loss of function of the PHYF protein obviating the need to measure reduction of mRNA or protein abundance in the mutants.

### PHYF plays a role in plant height during photomorphogenesis

**Figure 2:**
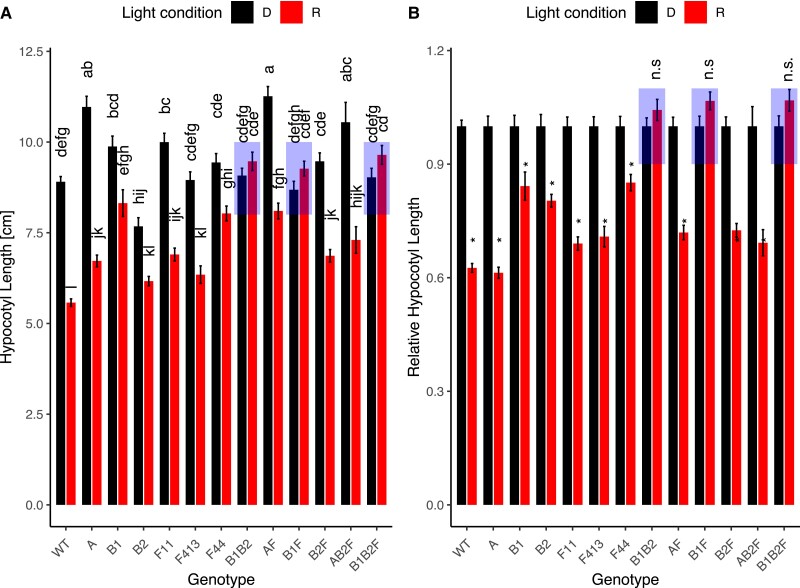
Loss of phyF when combined with loss of phyB1 affects the reduction of hypocotyl growth during photomorphogenesis in Rc. Seeds were germinated in the dark for 3–4 days. Synchronously germinated seedlings were transferred to R and allowed to grow for 4 days in Rc (15 µE), before being photographed, and analyzed using ImageJ as described in the Methods. Data were statistically analyzed using 2-way ANOVA, which showed a significant effect of interaction between genotype and light condition on hypocotyl length (*P* < 0.001). The data were subsequently analyzed with a Tukey post hoc test. Means not connected by the same letter are statistically significantly different from each other at *P* < 0.05. An asterisk indicates statistical significance at *P* < 0.05 from the dark treatment. For each genotype, four biological replicates were performed with similar results and data were pooled for this figure. Sample sizes were as follows (dark/red): A (*phyA*) = 112 (55/57), AB2F (*phyAB2F*) = 40 (16/24), AF (*phyAF*) = 122 (62/60), B1 (*phyB1*) = 91 (41/50), B1B2 (*phyB1B2*) = 99 (50/49), B1B2F (*phyB1B2F*) = 102 (55/47), B1F (*phyB1F*) = 96 (48/48), B2 (*phyB2*) = 107 (53/54), B2F (*phyB2F*) = 40 (17/24), F11 (*phyF-11*) = 113 (59/54), F413 (*phyF-413*) = 96 (46/50), F44 (*phyF-44*) = 119 (57/62), Wild-type cv. Moneymaker (WT) = 295 (141/154). Error bars reflect SE. The genotype *phyB1B2F* contained a mutation in a presumably unrelated second gene. A: shows data as absolute values; B: shows data as values relative to the dark response. Both A and B use the same data set. Shaded boxes are used to highlight the only three genotypes not responding to Rc. Rc = continuous red light; D = dark, R = red light; n.s. = not significant.

To determine what role phyF plays in the regulation of hypocotyl growth inhibition in response to R during photomorphogenesis, we incubated seeds in the dark and then transferred only synchronously germinated seedlings to experimental conditions. Despite slight variations in response, all single mutants, including the three *phyF* alleles, displayed a statistically significant reduction of hypocotyl growth in Rc compared to isogenic seedlings kept in the dark. These data show that loss of phyA, phyB1, phyB2, or phyF alone did not abolish growth inhibition in R (Figure 2). WT and *phyA* seedlings in R were reduced in height by 39% compared to those grown in darkness. Growth reduction in the *phyB1, phyB2,* and *phyF* alleles varied between 15%—31% (Figure 2). The *phyAF* double mutant growth reduction of 28% was similar to the responses of each of the two single mutants. Only seedlings with mutations in both *phyB1* and *phyB2,* or mutations in both *phyB1* and *phyF* resulted in the complete loss of R-light responsiveness producing essentially equally tall hypocotyls in Rc as in darkness (Figure 2). These data suggest that phyB1 can function without phyB2 or phyF to suppress hypocotyl growth while phyB2 function requires either phyB1 or phyF, and that phyB2 or phyF cannot function alone. Together these data are consistent with the notion that phyB2 and phyF cannot form functional homodimers.

### The response to photoperiod is redundantly regulated by phyB1 and phyF

**Figure 3:**
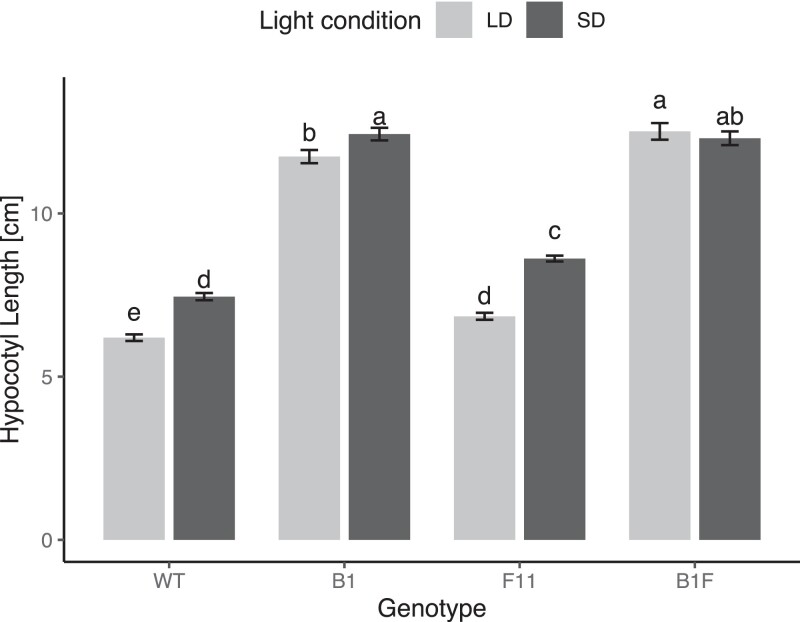
The photoperiod response in three-week-old seedlings is mediated redundantly by phyB1 and phyF. Seeds were germinated in darkness for 3–4 days and synchronously germinated seedlings transplanted and grown in experimental conditions for another 7 days. To ensure that only the photoperiod, and not also irradiance differed between treatments, the light intensity in the two conditions was adjusted such that seedlings experienced a similar total irradiance over a 24 h time period. Ten-day-old seedlings were measured using ImageJ. Two-way ANOVA showed a significant effect of interaction between photoperiod and genotype on hypocotyl growth (*P* < 0.001). Subsequently, a Tukey post hoc test was performed. Means not connected by the same letter are statistically significantly different from each other at *P* < 0.05. For each genotype, at least four biological replicates were performed and data were pooled for this figure. Sample sizes were as follows (LD/SD): B1 = 179 (94/85), B1F = 124 (64/60), F11 = 190 (88/102), WT = 204 (105/99). Error bars reflect SE. LD = long days, SD = short days. Gene abbreviations are as in Figure 2.

Wild-type Moneymaker tomato seedlings show distinct growth patterns in SD versus LD, where growth in SD leads to longer hypocotyls even if the total irradiance in both conditions is identical (Figure 3, Supplemental Figure 1). To test if phytochrome plays a role in this regulation, we grew seedlings mutant in phyB1, phyF-11, and phyB1/phyF-11 in both SD and LD R conditions and compared their hypocotyl growth to that of WT plants. Unsurprisingly, *phyB1* and *phyB1/phyF* mutants were overall taller than WT plants as were *phyF-11* single mutants compared with WT in each respective condition. Interestingly, single mutants retained a statistically significant phenotypic difference between growth in LD versus SD, however, plants mutant in both phyB1 and phyF appeared to be insensitive to the difference in day length. This suggests that phyB1 and phyF act additively in night-length sensing in tomato.

### Hypocotyl elongation reduction in FR does not require phyF

**Figure 4:**
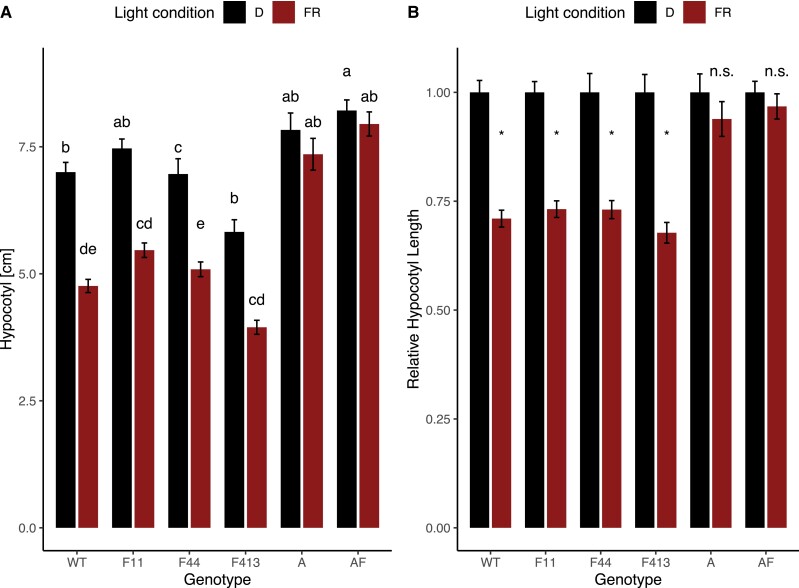
Unlike PHYA, PHYF is not required for the response to FRc light. Seeds were germinated in the dark for 3–4 days. Seedlings were selected for synchronous germination and transferred to experimental conditions for four additional days as described in the Methods and measured using ImageJ. A two-way ANOVA showed a significant effect of interaction between genotype and light condition on hypocotyl length (*P* < 0.001). Tukey post hoc analysis was subsequently performed. Means not connected by the same letter are statistically significantly different from each other at *P* < 0.05. An asterisk indicates statistical significance at *P* < 0.05 from the dark treatment. For each genotype, at least three biological replicates were performed and data pooled for this figure. Sample sizes were as follows (dark/far-red): A = 71 (38/33), AF = 84 (46/38), F11 = 105 (53/52), F413 = 81 (38/43), F44 = 90 (40/50), WT = 106 (55/51). Error bars reflect SE. Gene abbreviations are as in Figure 2. A: shows data as absolute values; B: shows data as values relative to the dark response. Both A and B use the same data set. FRc = continuous far-red light; D = dark, FR = far-red light; n.s. = not significant.

Close phylogenetic relationships between genes can suggest similarity in function. Given its sequence similarity with phyA, we tested if phyF plays a role in the perception and response to FR during seedling development. All three *phyF* alleles showed statistically significant shortening of the hypocotyl when exposed to FRc, while *phyA* mutants, as well as the *phyA/phyF* double mutants displayed no statistically significant difference in their response from plants grown in darkness (Figure 4). These data suggest that phyA, but not phyF, is the major FR receptor in 1-week-old hypocotyls.

### Far-red reversibility is modulated by phyA without the need of phyF

**Figure 5:**
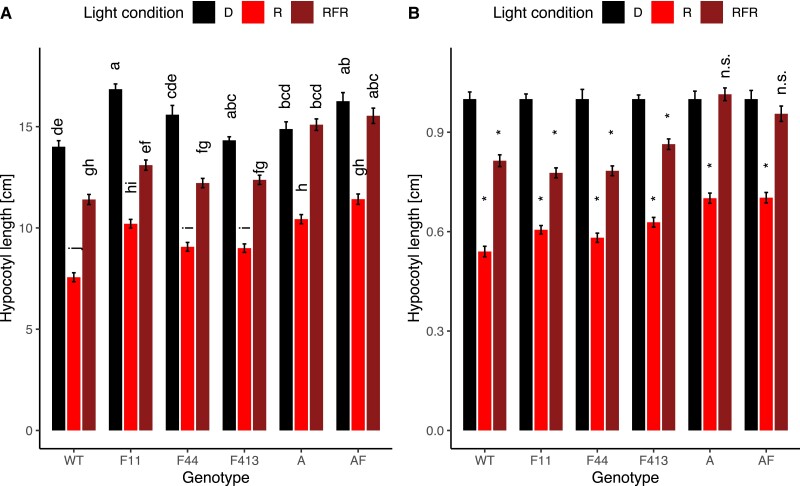
PHYA, but not PHYF, is required for FR reversibility to a R pulse. Seeds were germinated in the dark for 3–4 days. Synchronously germinated seedlings were transferred to experimental conditions. Seedlings were treated with pulses of R, R followed by FR, or kept in the dark, as described in the Materials and Methods. After four days in experimental conditions, plants were photographed and hypocotyl lengths measured using ImageJ. A 2-way ANOVA showed a significant effect of interaction between genotype and light condition on hypocotyl growth (*P* < 0.001). Subsequently, a Tukey posthoc test was performed. Means not connected by the same letter are statistically significantly different from each other at *P* < 0.05. An asterisk indicates statistical significance at *P* < 0.05 from the dark treatment. For each genotype, at least four biological replicates were performed and data pooled for this figure. Sample sizes were as follows (dark/red/red + far-red): A = 130 (42/44/44), AF = 133 (43/49/41), F11 = 122 (39/42/41), F413 = 124 (40/43/41), F44 = 122 (36/48/38), WT = (41/54/42). Error bars reflect SE. Gene abbreviations are as in Figure 2. A: shows data as absolute values; B: shows data as values relative to the dark response. Both A and B use the same data set. D = dark, R = red light; RFR = red light followed by a far-red pulse; n.s. = not significant.

For many but not all physiological functions, R-induced phytochrome action can be reversed by illumination with FR (Mancinelli, 1994). Reversibility usually requires FR irradiation within a short period of time from R irradiation to avoid escape of the R-mediated response. We asked if phyF is required for reversibility of the hypocotyl growth inhibition response. We exposed synchronously germinated seedlings to pulses of R or R followed by FR as described in the Methods and measured hypocotyl length after 96 h. The effect of light treatment on hypocotyl length depended on the genotype (2-way ANOVA, *P* < 0.05, Figure 5). We followed up the 2-way ANOVA with Tukey post hoc analysis. The data showed that, similar to Figure 2, removal of phyA or phyF did not result in a loss of responsiveness to R. When treated with FR after exposure to a R pulse, WT plants showed an intermediate phenotype that was between growth in darkness and growth in R-pulsed seedlings. Loss of phyF did not change the reversibility response compared with the WT, however, loss of only phyA was sufficient to completely reverse the R signal by FR treatment back to the dark response (Figure 5). Deletion of both phyA and phyF in the double mutant showed no difference from the *phyA* single mutant response (Figure 5).

### PHYF participates in the end-of-day FR (EODFR) response

**Figure 6:**
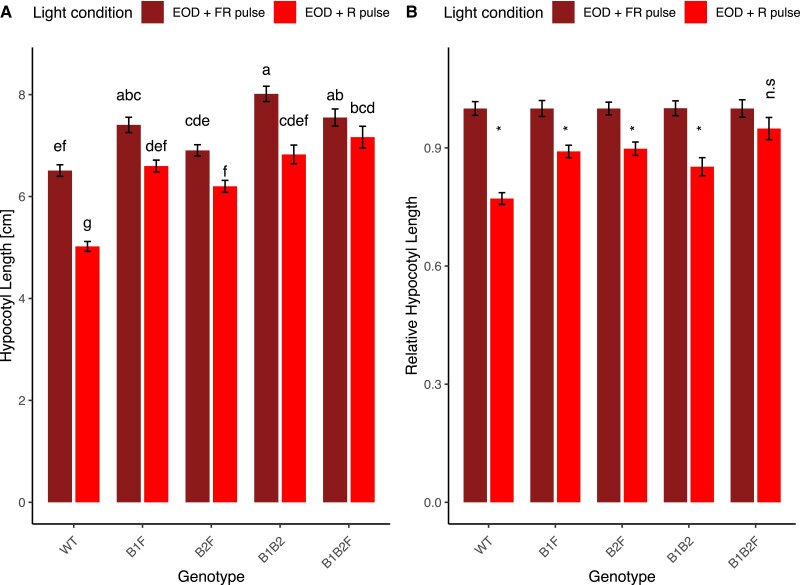
PhyF plays a role in the response to end-of-day treatment with FR. Seeds were germinated in darkness for 3–4 days and synchronously germinated seedlings transplanted and grown in experimental conditions for an additional four days. Seedlings were then measured using ImageJ. Two-way ANOVA showed a significant effect of interaction between light treatment and genotype on hypocotyl growth (*P* < 0.001). Subsequently, a Tukey post hoc test was performed. Means not connected by the same letter are statistically significantly different from each other at *P* < 0.05. An asterisk indicates statistical significance at *P* < 0.05 from the dark treatment. For each genotype, five biological replicates were performed and data were pooled for this figure. Sample sizes were as follows (EOD + FR/EOD + R): B1F = 115 (45/70), B2F = 121 (68/53), B1B2 = 132 (78/54), B1B2F = 128 (63/65), WT = 151 (75/76). Error bars reflect SE. The genotype *phyB1B2F* contained a mutation in a presumably unrelated second gene. Gene abbreviations are as in Figure 2. A: shows data as absolute values; B: shows data as values relative to the dark response. Both A and B use the same data set. EOD + FR pulse = end-of-day plus FR treatment, EOD + R = end-of-day plus R treatment. FR = far-red light; R = red light; n.s. = not significant.

Plants mutant in both phyB1 and phyB2 were previously reported to show residual responses in hypocotyl and internode growth under low R:FR compared with high R:FR light conditions (Schrager-Lavelle et al., 2016). To test if phyF has residual function in the end-of-day FR response we compared hypocotyl elongation between the various double and triple mutant combinations involving phyB1, phyB2, and phyF. As expected from the literature, *phyB1/phyB2* double mutants responded to EODFR treatment with a robust increase in hypocotyl length compared to EODR treatment (Figure 6) as did both double mutants containing the *phyF-11* allele in either the *phyB1* or *phyB2* background. However, hypocotyls of the *phyB1/phyB2/phyF* triple mutant displayed statistically insignificant differences under EODFR conditions (Figure 6), suggesting that phyF indeed plays a role in this response.

*Both* phyA *and* phyF *affect root length in a synergistic manner*

**Figure 7:**
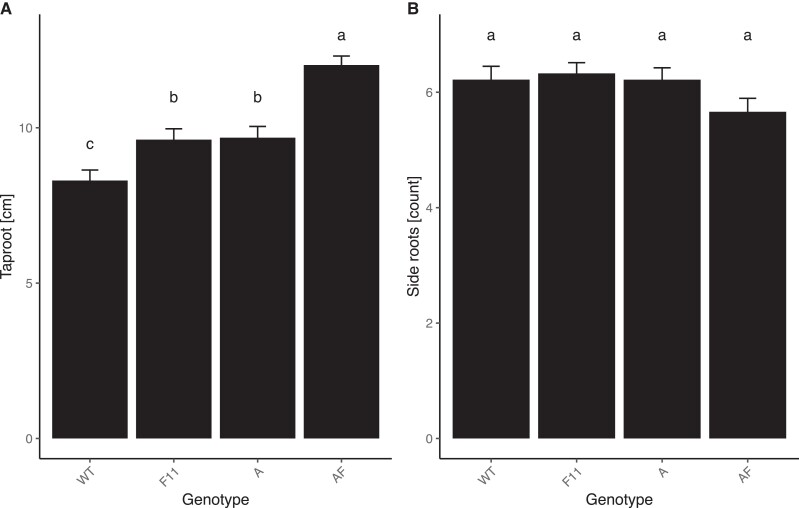
Taproot length is additively regulated by both phyA and phyF. Seeds were germinated and grown in vermiculite soaked in Hoagland solution and grown for 3 weeks. Roots were photographed and analyzed using ImageJ. A: The length of the longest taproot was measured and analyzed using one-way ANOVA (*P* < 0.001) followed by Tukey post hoc analysis. B: The total number of side roots were counted and analyzed by a one-way ANOVA (*P* = 0.116), showing they did not differ between genotypes. Means not connected by the same letter are statistically significantly different from each other at *P* < 0.05. Sample sizes for both experiments were as follows: A = 54, AF = 64, F11 = 54, WT = 64. Error bars reflect SE. Gene abbreviations are as in Figure 2.

To determine if phyF is involved in below-ground responses, we grew plants in white light in vermiculite for three weeks and then measured the length of the taproot and counted the number of lateral roots. Taproot length was significantly increased in both single mutants. Interestingly, double mutants had even longer roots than both of the single mutants (1-way ANOVA, *P* < 0.001, Figure 7A), suggesting that phyA and phyF can act synergistically in repressing taproot growth. Lateral root number, on the other hand, was unaffected by mutations in phyA or in phyF, as well as in the *phyA/phyF* double mutant (1-way ANOVA, *P* > 0.05, Figure 7B).

### No evidence that phyF interacts with blue light signaling or is singularly required in adult vegetative tissue development

To determine if phyF plays a role in synergistic interactions with blue light receptors or blue light signaling, we germinated WT and all three phyF mutant alleles in darkness before transferring synchronously germinated seedlings to agar filled Magenta Jars and exposing them to either R or R + B for 3 days. All red light-treated genotypes were more than twice as tall as their isogenic R + B-treated counterparts (two-way ANOVA and Tukey post hoc, *P* < 0.05). However, there was no difference (two-way ANOVA and Tukey post hoc, *P* > 0.05) in hypocotyl length between the WT and any of the three *phyF* alleles, which ranged in height from 2.8 cm to 3.0 cm, in the R + B light. These data suggest that in the conditions tested, phyF is not required for interacting with the B-sensing pathways in tomato (Supplemental Figure 2).

We also investigated the effect of the *phyF* mutation on internode length of the first few internodes measured at weeks 4 and 6, as well as on flowering time and leaf blade length (see Supplemental Methods) but failed to detect any statistically significant differences in these phenotypes (Supplemental Figures 3-5).

## Discussion

Functional phytochrome analysis has made frequent use of loss-of-function mutants in Arabidopsis, tomato, and other species. In Arabidopsis, mutants are available for each phytochrome gene and for higher order mutants. In tomato until now stable knockout mutants were only available for *SlPHYA*, *SlPHYB1*, and *SlPHYB2* (van Tuinen et al., 1995a; van Tuinen et al., 1995b; Weller et al., 2000). A knock-down mutant with 50% efficiency has also been described for *SlPHYE* (Schrager-Lavelle et al., 2016). With this work, by creating a series of knockout alleles, we are adding additional mutants to the collection and have begun to describe the role phyF plays in plant growth and development in tomato.

Our data show that mutation of SlphyF in a *phyB1* mutant background leads to the complete loss of R-responsiveness during photomorphogenesis (Figure 2). Previous work (Weller et al., 2000) had shown that *phyA,* and *phyB1* seedlings grown for 12d in low intensity (3 µmol*m^−2^*s^−1^) continuous broadband R were taller than WT seedlings in R, while *phyB2* showed no difference from the WT. At the same time, each of the *phyA, phyB1* and *phyB2* single mutants, as well as the *phyA/phyB1* and the *phyA/phyB2* double mutants were shorter in R than the WT in darkness (Weller et al., 2000), suggesting that no single phytochrome gene was alone responsible for the response. It was less clear in the previous work by Weller and colleagues (2000) if the *phyB1/phyB2* double mutant showed any residual R-induced hypocotyl growth suppression response. Our data largely corroborate the previous findings and add the observation that loss of phyF by itself also does not abolish a statistically significant R-light response. But while the combined loss of phyA and phyB1, as well as the combined loss of phyA and phyB2 still allows for a strong reduction in the R response (Weller et al., 2000), combined loss of phyB1 with phyF abolishes the response completely (Figure 2). In our hands, loss of both *phyB1* and *phyB2* in the respective double mutant also eliminated the response in a statistically significant manner. Notably, the double mutant between *phyB2* and *phyF* had a strong R response, showing that another phytochrome, presumably phyB1, can facilitate the response in the combined absence of phyB2 and phyF. In Arabidopsis, heterodimerization of AtphyB and AtphyC has been shown to occur in vitro (Sharrock and Clack, 2004) and in planta (Sánchez-Lamas et al., 2016), resulting in strong nuclear localization of the heterodimer. Our data suggest that in tomato heterodimers of phyB1 with either phyF or phyB2, heterodimers of phyF with phyB2, or phyB1 homodimers are required and sufficient to effectively reduce hypocotyl elongation in R.

*AtphyC* seedlings are somewhat longer than WT but much shorter than *AtphyB* when grown in R and compared with WT (Monte et al., 2003). Our data (Figures 2 and 4) show that the *phyF* mutants are slightly but significantly taller than WT plants in R regardless of day length. This might either suggest a role for phyF in hypocotyl elongation suppression even without the requirement of interaction with phyB1 in response to the R spectrum, or interaction between R and B light signaling. However, our experiments in combined R + B conditions showed no difference between the three *phyF* alleles and the WT response providing no immediate indication that phyF interacts with the B pathways (Supplemental Figure 2).

**Figure 8:**
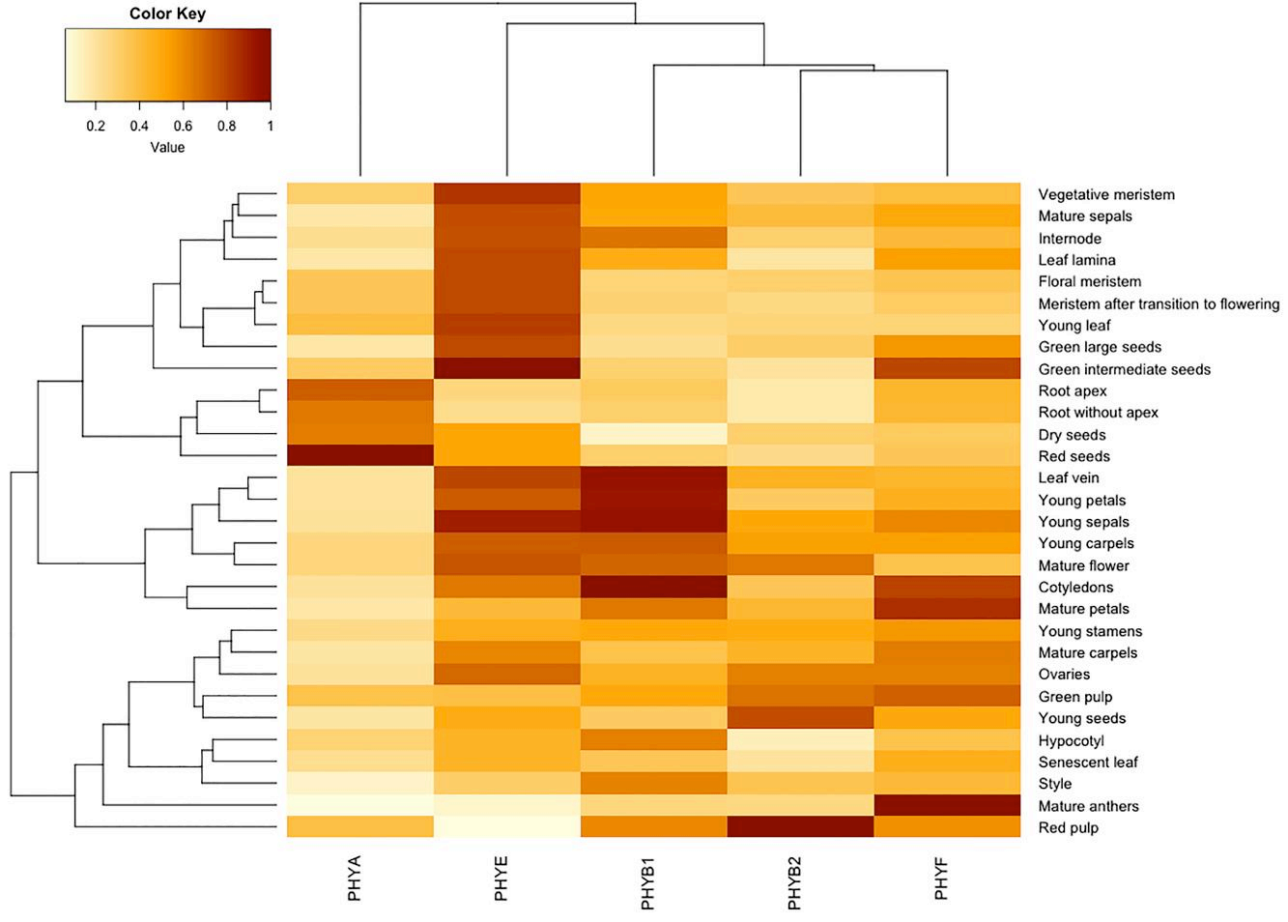
PHYF is expressed weakly to moderately in hypocotyls and other young tissue but is more strongly expressed in older tissues. Data were mined from the Transcriptome Variation Analysis database http://travadb.org/and visualized using R software. Each gene's expression patterns are normalized against their highest expression value (darkest shade of red). Lower expression levels correspond to lower color values/lighter shades/yellow, higher expression levels correspond to higher color values/darker shades/red-brown.

Phytochrome C has been implicated in flowering time regulation in Arabidopsis and wheat. In Arabidopsis, AtphyC promotes flowering in LD redundantly with AtphyA (Monte et al., 2003), but is required for controlling flowering time in SD (Monte et al., 2003). By contrast, in LD-flowering tetraploid wheat (*Triticum turgidum*) the TtphyC homolog was required for acceleration of flowering (Chen et al., 2014), while in the SD flowering plant rice (*Oryza sativa*) loss of phyC in LD led to somewhat accelerated flowering in non-inductive LD photoperiods (Takano et al., 2005). In tomato, we did not find any significant differences in flowering time between WT and *SlphyF* mutants in tomato (Supplemental Figure 3). It is possible that this is due at least in part to the fact that cultivated tomato is a day neutral plant where day length does not affect flowering time the same way as it does in Arabidopsis (Lifschitz and Eshed, 2006; Soyk et al., 2017). Given this difference in phyC/F involvement in flowering between tomato on the one hand and Arabidopsis, bread wheat (*Triticum aestivum*) and rice on the other hand, along with the observation that SlphyF expression is nonetheless strongest in tomato flowers (Figure 8), more work is currently in progress to further investigate SlphyF's role in flower development.

Previous work has shown that growth in short days leads to taller hypocotyls compared with growth in long days, both in Arabidopsis and in tomato (Niwa et al., 2009; Hwang et al., 2020). In Arabidopsis, photoperiod-dependent hypocotyl elongation is modulated by PHYTOCHROME INTERACTING FACTORs (PIFs) 4 and 5 (Niwa et al., 2009). Given that *AtPIF4* and *AtPIF5* duplicated after the split of the Brassicaceae from the Solanaceae (Rosado et al., 2016), and given that in tomato phyB1 and phyF are required for the photoperiodic response to R (Figure 3), it seems plausible that SlPIF4 may interact with phyB1 and phyF, maybe in a phyB1/phyF heterodimeric form, in modulating the response in tomato.

In Arabidopsis, germination and de-etiolation in low light conditions are known to be mediated by phyA's ability to detect and respond to very low fluence FR, allowing for example for activation of germination even in low light conditions or detecting early light at dawn (Seaton et al., 2018) in what is called the very low fluence response (VLFR). SlphyF is phylogenetically more closely related to phytochrome SlphyA than to the other phytochromes SlphyB1, SlphyB2, and SlphyE (Alba et al., 2000), suggesting that functionally some overlap might exist between the two genes. On the other hand, hierarchical clustering of temporal transcriptional activity (Figure 8) showed that *SlPHYA* and *SlPHYF* peak activities do not overlap much throughout development. Our data show a complete reversal of the R-induced response after pulsing with FR only in both *phyA* and *phyA/phyF* mutants (Figure 5). This suggests that FR successfully inactivates phyF. By contrast, our data are consistent with the notion that FR does not fully reverse the Pfr form of phyA, which then triggers a VLFR response in WT and each of the phyF alleles—but not in individuals carrying a mutant phyA allele (Figure 5). It is of note, that this presumably phyA-induced hypocotyl shortening response results in hypocotyl length that is intermediate to both the R-induced hypocotyl inhibition and the etiolated growth in the dark. Loss of phyA, as seen in the single and double mutants, however, genetically removes the single phytochrome that remains active after FR treatment, leading to a response that is identical to growth in the dark (Figure 5). These data are similar to previous findings by van Tuinen and colleagues (1995a), who showed that FR in *phyA* mutants is still able to return activated phytochrome into the inactive Pr form, although their data were somewhat ambiguous about the magnitude of the R/FR response vis-à-vis the dark grown control. Our data largely confirm these previous findings and add that despite its phylogenetic distance to phyA, phyF has the hallmarks of a typical FR-reversible phytochrome with respect to low fluence FR induced reversal during photomorphogenesis.

To further define the role that phyF plays in R/FR sensing we measured hypocotyl lengths in short days followed by EOD pulses of either FR or R. Under these conditions, the FR treated plants should have low concentrations of active Pfr during the night, while the control plants treated with the EODR pulse should have high levels of Pfr. As shown previously, *SlphyA* mutants and wild-type plants have similar responses to EODFR treatment, suggesting that phyA plays no or only a small role in these conditions in tomato (van Tuinen et al., 1995a). In EODFR conditions, we argued, any possibly remaining R/FR responses in the *phyB1/phyB2* double mutant could thus be ascribed to phyE or phyF activity. Our data show a significant difference between the EODFR and EODR response in the *phyB1/phyB2* double mutant but only a small, statistically no longer significant difference in the triple *phyB1/phyB2/phyF* mutant (Figure 6), suggesting that phyF is involved in EODFR sensing. Previous work using an artificial miRNA *phyE* knock-down mutant had shown a small, but statistically significant role for tomato phyE in the shade avoidance response in 5-week-old seedlings (Schrager-Lavelle et al., 2016). Our data suggest a role for phyF in R/FR detection responses and leave open the possibility that phyE also plays a small, maybe additive role in the EOD response in younger seedlings. A currently unavailable quadruple knockout mutant of phyB1, phyB2, phyE, and phyF could help shed further light on this question.

Interestingly, root growth appeared to be additively controlled by phyA and phyF (Figure 7). Involvement of different phy in root development in Arabidopsis and *Nicotiana attenuata* under various conditions has been described in the past (Correll and Kiss, 2005; Costigan et al., 2011; Oh et al., 2018). We also previously showed that in 5d-old tomato WT seedlings, phyA reduces root length in R (Carlson et al., 2019). Our data in three-week-old plants grown in white light support these findings (Figure 7A). The additive role that phyF and phyA play in root patterning deserves further investigation (Figure 7A).

PHYF is generally more transcriptionally active in older tissues than in younger ones, although mRNA was reported in all tissues that were sampled (Figure 8). PhyF appears to play important roles in more mature tissues, especially in anthers (Figure 8). We grew WT and mutants to adulthood and assayed a variety of vegetative phenotypes. No statistically significant differences were observed in leaf length (Supplemental Figure 4), although phyF transcription in WT is relatively high in that tissue (Figure 8). No differences were also found in the length of any of the most basal six internodes in 3-week and 6-week-old plants (Supplemental Figure S5). Internode length in tomato is known to be regulated by cryptochromes 1 and 2 (cry1/cry2), which redundantly shorten especially the lowest internodes in response to B or white light (Fantini et al., 2019). Our adult vegetative phenotype findings suggest that phyF either does not play an important role in these tissues at the sampled developmental stages, or that other phytochromes provide redundant functionality.

### Conclusions and outlook

Creating a set of mutants allowed us to begin to show that phyF in tomato plays roles in early photomorphogenesis, EODFR- and photoperiod sensing. Additional work will be needed to assess the validity of these interpretations. Ongoing work is currently directed at understanding phyF's role in flower development and its involvement in coordinated responses with the other phytochromes in tomato.

## Materials and methods

### Mutant construction

A vector, carrying the target sequence, was built for the purpose of CRISPR-Cas9 mutagenesis (Figure 1) using previously established methods in tomato (*Solanum lycopersicum*) (Brooks et al., 2014). The vector was introduced into *Agrobacterium tumefaciens* (strain LBA4404) and transformed into tomato variety Moneymaker by the Van Eck group at the Boyce Thompson Institute. The presence of the T-DNA construct indicating successful transformation was validated in the first generation transgenic plants (T0) by PCR using primers targeting the 35S promoter and Cas9 sequence (Supplemental Table 1). *PHYF* genes from the T0 plants were then amplified with PCR, cloned, and sequenced (Supplemental Table 1) to identify those with targeted mutations. Every plant that was successfully transformed with Cas9 and guide RNAs (gRNAs) showed mutations in the target genes, whereas those control plants transformed with Cas9 and no guide RNAs showed no mutations in the target genes. T0 plants were grown to adulthood, allowed to self, and T1 offspring from each individual were planted and screened. The T1 plants were first screened for the presence of the T-DNA construct as in the T0 (Supplemental Table 1). Those without evidence of the T-DNA, on average one in four offspring for single insertion events or one in 16 for double insertions, were genotyped for their targeted mutations. Three *phyF* lines (*phyF-11, phyF-44, phyF-413*) were identified without the T-DNA construct and with targeted mutations. All of these mutations lead to predicted truncated proteins due to early stop codons. Confirmed mutant lines were propagated by selfing (in the case of *phyF-11* another round of backcrossing and mutant recovery was added), and subsequently used for seed bulking. The triple mutant *phyB1/B2/F* contains an additional mutation in a presumably unrelated, gravitropic response gene (*lazy-2*), that was inadvertently introduced in this line during higher order mutant construction.

### Plant materials and growth conditions

*Solanum lycopersicum* seeds of cultivar Moneymaker [original source: Tomato Genome Resource Center (TGRC), Davis, CA, United States] and homozygous *phyF* mutants (alleles *phyF-11, phyF-44, phyF-413)* were used in the experiments as indicated. A double mutant for phyA and phyF was created by cross-pollination of the homozygous *phyA* mutant *(fri*) in the Moneymaker background (Tomato Genome Resource Center, Davis, CA, United States) and *phyF-11*. The *phyB1* (allele tri^1^) and *phyB2* (allele 70F) single mutants, and the *phyB1phyB2* double mutant (with the tri^1^ and 70F mutant alleles, respectively) were originally obtained from the TGRC. All higher order mutants containing *phyB1* or *phyB2* also use the same alleles; higher order mutants with *phyF* contain the *phyF-11* allele. The *phyA, phyB1,* and *phyB2* mutants were described previously (van Tuinen et al., 1995a; Weller et al., 2000). For physiological seedling experiments, seeds were surface sterilized with 50% (v/v) bleach for 15 min in ambient lab conditions and then sown on water-saturated paper towels in light-excluding plastic boxes. Plants were germinated in a dark growth chamber at 25°C. To ensure synchronously germinated plants, seedlings for all photomorphogenesis experiments were selected when they were ∼ 2 cm tall (after ∼3–4 days), and transferred before treatment from the germination boxes to 7.6 × 7.6 × 10.2 cm clear Magenta Jars where they were grown for an additional 4 days (R experiments, FR experiments, and pulse experiments) or an additional 7 days (photoperiod experiments). The jars contained ∼100 ml of 1% (w/v) agar growth medium supplemented with 1.87 g/L (0.5X) Murashige and Skoog salts (Sigma) pH 5.8, and were sealed with a lid.

### Light treatment

For seedling phenotypes in continuous red light (Rc), after dark germination, seedlings were kept in temperature-controlled incubators and illuminated with light from a 25W red LED bulb (ABI LED lighting, abilights.com) with an emission spectrum maximum of 660 nm and a total PPF of ∼15 µmol*m^−2^*s^−1^ at seedling level for 3 days.

Synchronously germinated seedlings used for the continuous far-red (FRc), R/FR reversal, and end-of-day far-red pulse experiments were kept in growth chambers for 3 days at 25°C and illuminated with either one 25W R LED bulb (ABI LED lighting, abilights.com, emission spectrum maximum of 660 nm) or a 16W FR LED bulb (Agromax, www.htgsupply.com) with an emission spectrum maximum of 730 nm and a light intensity of ∼12 µmol*m^−2^*s^−1^ at plant level. Light intensities were measured using an SS-110 field spectroradiometer (Apogee Instruments). The lamps were controlled by electronic timers that were adjusted as needed.

For photoperiod experiments, plants were either grown in red light in long days (LD) with 16 h/8 h light/dark periods, or in R in short days (SD) with 8 h/16 h light/dark periods in an incubator set at continuous 25°C. Lights were fitted with 660 nm red LED bulbs (ABI LED lighting, abilights.com). The light intensity in the SD chamber was ∼31 µmol*m^−2^*s^−1^. The light intensity in the LD chamber was reduced to ∼15 µmol*m^−2^*s^−1^ by filtering the light through a 50% neutral density filter. After germination and selection, plants were kept in continuous light conditions at 25°C for 3 days.

Seedling responses to R/FR pulse illumination were analyzed as follows: After transfer to Magenta Jars, seedlings were kept in darkness, except when pulsed with light. Plants were either treated only with R-light pulses (3 min every 4 h) or with pulses of 3 min R followed by 6 min of FR light every 4 h for a total of 4 days.

For the end-of-day far-red (EODFR) treatment, synchronously germinated seeds were transferred to one of two Percival incubators (Percival Scientific Inc.) fitted with fluorescent white light at an intensity of ∼ 75 μmol/m^2^/s and a R/FR ratio of ∼ 1.75 in 8 h/16 h light/dark cycles and kept in those conditions for 4 additional days. At the end of each light cycle, plants were either treated with 15 min of saturating pure R or pure FR light from the same LED light sources described above.

Blue light experiments were performed using blue (460 nm) and red LEDs (660 nm), or red LEDs alone in a Percival LED 30L-1 chamber. The light intensity in the R only conditions was ∼30 µmol*m^−2^*s^−1^, the intensity in the B + R chamber was ∼118 µmol*m^−2^*s^−1^ with a B:R of 3.4. After germination and selection, plants were kept in continuous light conditions at 25°C for 3 days.

For bulking and higher order mutant construction, plants were grown in a greenhouse under lighting conditions that varied throughout the year, but days were extended to 16 h with sodium lamps and R/B/W LED arrays (BESTVA 2000W, LM301B). Spectral profiles of the light sources used were measured using a spectroradiometer (SS-110, Apogee Instruments, apogeeinstruments.com) and can be found in Supplemental Figures S6-S8.

### Seedling phenotype growth analysis

Seedlings were removed from their growth containers at indicated times and photographed. Digital images were used to measure hypocotyl and root lengths using the analysis program ImageJ (https://imagej.nih.gov/ij/).

### Root phenotypic analysis

Seeds were sterilized in 50% (v/v) household bleach for 15 min. Seeds were planted in plant trays 1–2 cm deep in vermiculite saturated with Hoagland solution (1.63 g/L). The trays were covered with clear plastic wrap and incubated at 22°C with 16 h/8 h light/dark cycles using fluorescent lights at a light intensity of ∼ 83 µmol*m^−2^*s^−1^ and a R/FR ratio of 1.3. The plastic wrap was removed when seedlings emerged and the vermiculite was watered daily (6 days/week) with 500 ml Hoagland solution, which was fully absorbed by the medium. At 21 days post-planting plants were removed from the trays, rinsed in water and placed on a dark surface in a shallow pool of water to spread out the roots. Plants were photographed and the pictures were analyzed with ImageJ.

### Statistical analysis

All statistical analyses were conducted with R version 3.5.0 (or higher).

### Accession numbers

Sequence data from this article can be found in the GenBank/EMBL data libraries under accession numbers NM_001247561.2 (PHYA), NM_001306202.1 (PHYB1), NM_001330171.1 (PHYB2), NM_001329760.1(PHYE), NM_001320517.1(PHYF).

## Supplemental data

The following materials are available in the online version of this article.

**Supplemental Methods S1.**

**Supplemental Figure S1.** Spectral profile of far-red light source used for all experiments involving far-red light.

**Supplemental Figure S2.** Spectral profile of red light source used for all experiments shown in Figures 2 and 4, 5, and 6.

**Supplemental Figure S3.** Spectral profile of red and blue light sources used in blue/red experiments shown in **Supplemental Figure S5**.

**Supplemental Figure S4.** Representative seedlings from the photoperiod experiments in Figure 3.

**Supplemental Figure S5.** The addition of continuous blue light (Bc) to continuous red light (Rc) does not elicit a differential response between WT and *phyF* mutants.

**Supplemental Figure S6.** PhyF does not appear to regulate flowering time significantly by itself.

**Supplemental Figure S7.** PhyF does not play a role in regulating leaf length or the function is fully redundant with at least one other phytochrome.

**Supplemental Figure S8.** PhyF is does not mediate internode elongation of internode 2 in weeks 4 and 6.

**Supplemental Table S1.** PCR primers for constructing and genotyping CRISPR-Cas9 mutated lines.

## Supplementary Material

kiad028_Supplementary_Data
