# Supplementary material for: Phytochrome F mediates red light responsiveness additively with phytochromes B1 and B2 in tomato
Source: Plant Physiol. 2023 Jan 21;191(4):2353–66. doi: 10.1093/plphys/kiad028 (PMC10069882; doi:10.1093/plphys/kiad028)
Supplement: kiad028_Supplementary_Data [file kiad028_supplementary_data.pdf]

SUPPLEMENTAL MATERIAL

Supplemental Methods

Phenotypic analysis in adult plants

MoneyMaker (MM) WT plants, along with the *phyF* alleles *phyF-11, phyF-44*, and *phyF-413* were plated directly in soil in 10 cm x 10 cm square pots. The potting soil (Sunshine Mix #4; Sungrow Horticulture) was water-saturated and mixed with Marathon 1% granular insecticide (Olympic Horticultural Products, Bluffton, SC) as per product instructions. Initially, 3 seeds were planted per pot and thinned to one per pot after germination. The seeds were planted by placing them on top of the soil, after which an approximately 5 mm thick layer of prepared soil was added over the top. Pots were randomly arranged in trays and grown for 4 weeks in an incubator (continuous 25°C, 16 h of white light/ 8 h of darkness per day). The pots were re-randomized weekly to prevent bias from any potential microclimate differences within the incubator. Water in the trays was topped off whenever the trays dried out, approximately every 2-3 days. After 4 weeks the plants were moved to the greenhouse. They were re-potted in 20-cm diameter, round, plastic pots, with the same soil mixture as described above and supplemented with a teaspoon (5 ml volume) of Osmocote soak-in fertilizer pellets (The Scotts Company, LLC), which were sprinkled on top of the soil. The pots were randomly arranged on a bench, and stayed in that arrangement for the remainder of the experiment. The plants were connected to an automatic watering system and received full sunlight during the day supplemented with two 400-W metal halide lamps and one 2000W LED full spectrum grow light (Bestva.com, DC series).

Flowering time was measured as the number of days to develop the first blossom with yellow petals, of which at least two petals were at least half extended from the closed position. In weeks three and six the bottom three or six internode lengths were measured, respectively, with a ruler. The leaf length of the bottom-most compound leaves of 4-week old plants was measured with a ruler from the base of the leaf where it meets the petiole to the tip of the terminal leaflet.

For each phenotype measured 1-way ANOVAs followed by Tukey post-hoc analyses were conducted. All analysis was conducted using R. The experiment was done in three biological replicates grown at different times of the year. Data were analyzed separately and pooled after ascertaining that results were similar between biological replicates.

Supplemental Figures

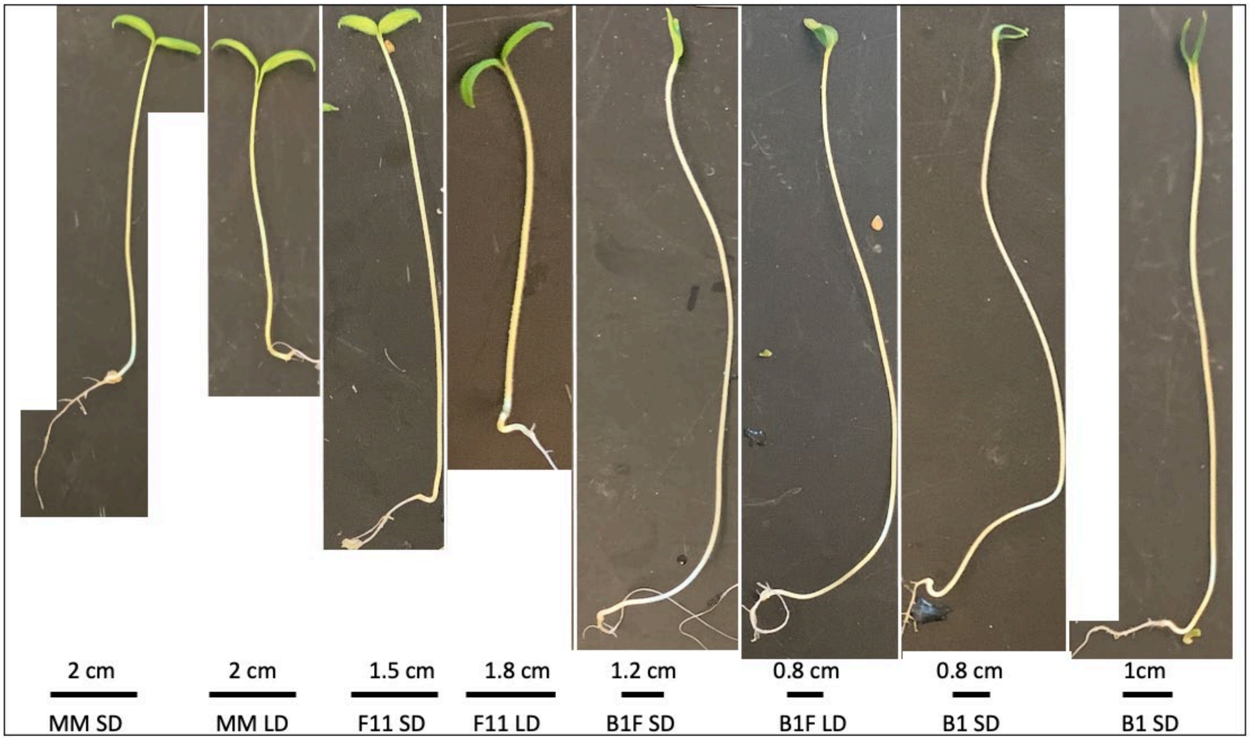

Supplemental Figure S1: Representative seedlings from the photoperiod experiments in Fig. 3. SD: short day, LD: long day. MM = Wildtype cv. Moneymaker, F11 = *phyF-11*, B1F = *phyB1F*, B1 = *phyB1*

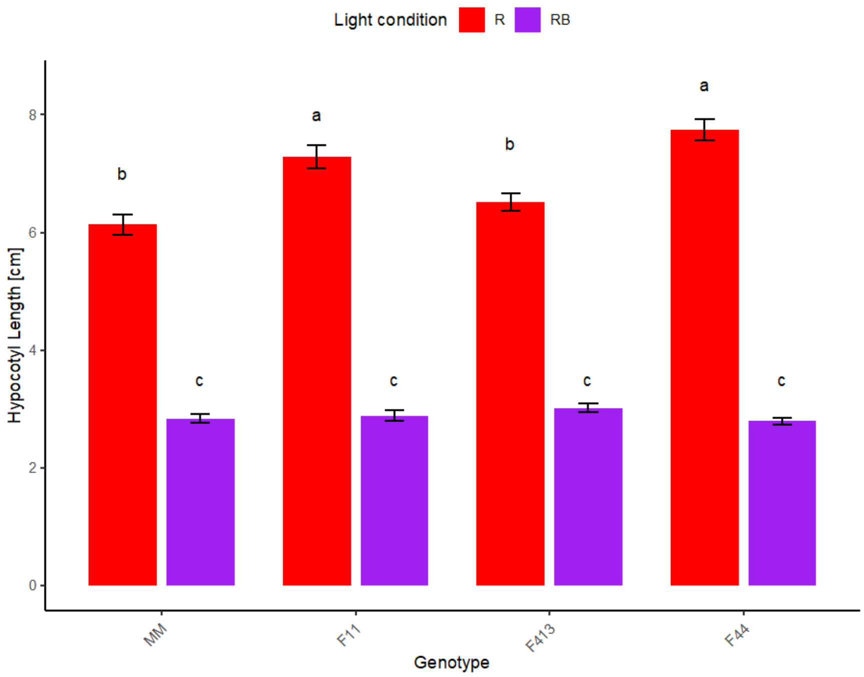

Supplemental Figure S2: The addition of continuous blue light (Bc) to continuous red light (Rc) does not elicit a differential response between WT and *phyF* mutants. Seeds were germinated in darkness for 3-4 days and synchronously germinated seedlings transplanted and grown in experimental conditions for another 4 days and subsequently measured using ImageJ. A Tukey posthoc test showed no significant effects of genotype on hypocotyl length in Rc+Bc ($p > 0.05$). Means not connected by the same letter are statistically significantly different ($p < 0.05$) from each other. For each genotype, at least four biological replicates were performed and data were pooled for this figure. Sample sizes were as follows (Rc/RcBc): MM (Wildtype cv. Moneymaker) = 119 (62/57), F11 (*phyF-11*)= 91 (48/43), F44 (*phyF-44*) = 110 (56/54), F413 (*phyF-413*) = 146 (77/69). Error bars reflect SE. Gene abbreviations are as in Fig. 2. R = continuous red, RB = continuous red + continuous blue.

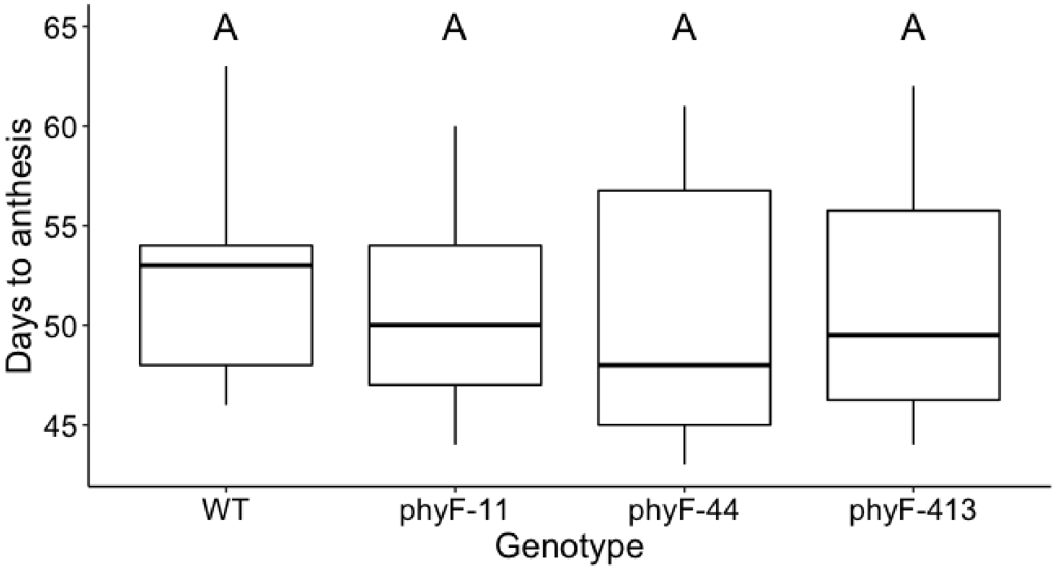

Supplemental Figure S3: PhyF does not appear to regulate flowering time significantly by itself. There is no statistically significant difference between any genotypes with respect to flowering time (ANOVA: $p = 0.836$, $F = 0.247$, $df = 3$, $N = 21$ per genotype except F413, for which there were 20). The data were combined from three separate biological replicates. The bold line in each box represents the median. Lower and upper box limits indicate the first and third quartiles. The whiskers extend from the top or bottom of the box to the largest or smallest value, but no further than 1.5 times the interquartile range. Outliers beyond the whiskers are indicated by individual points.

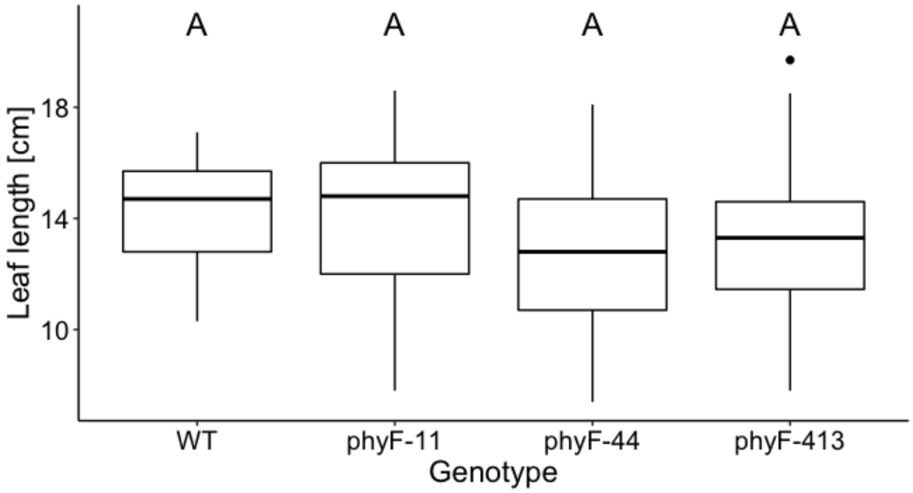

Supplemental Figure S4: PhyF does not play a role in regulating leaf length or the function is fully redundant with at least one other phytochrome. The bottom-most leaf of each plant was measured in 4-week old plants. There is no statistically significant difference between any genotypes with respect to leaf length (ANOVA: p = 0.198, F = 1.593, df = 3, N = 21 per genotype except F413, for which there were 20). The data were combined from three separate biological replicates. The bold line in each box represents the median. Lower and upper box limits indicate the first and third quartiles. The whiskers extend from the top or bottom of the box to the largest or smallest value, but no further than 1.5 times the interquartile range. Outliers beyond the whiskers are indicated by individual points.

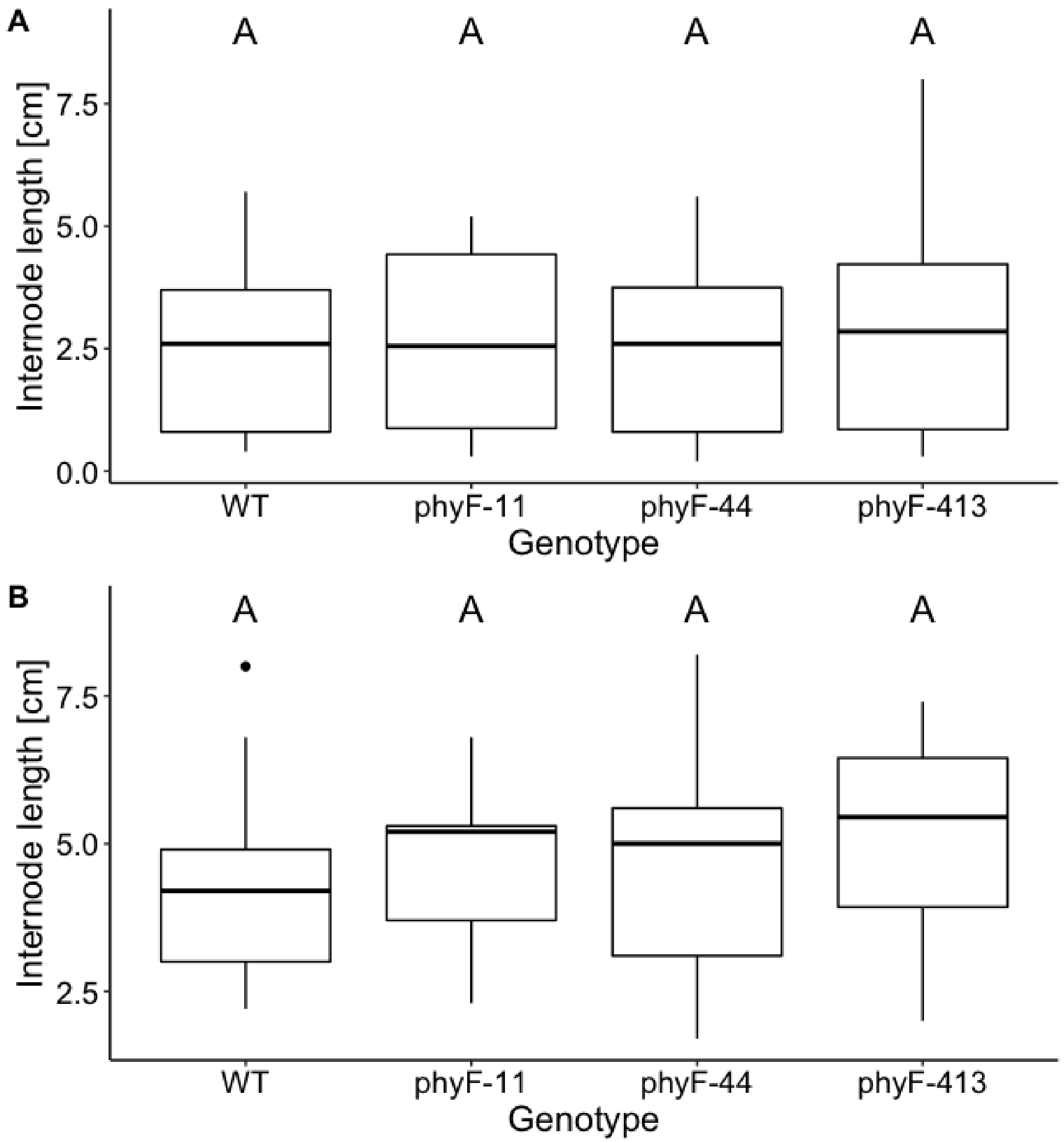

Supplemental Figure S5: PhyF is does not mediate internode elongation of internode 2 in weeks 4 and 6. (A) In 4-week old seedlings, no difference in length of internode 2 (counted from the base of the plant) was observed (ANOVA: $p = 0.939$, $F = 0.134$, $df = 3$, $N = 21$ per genotype except F413, for which there were 20). (b) In 6-week old seedlings, no difference in length in internode 2 was observed (ANOVA: $p = 0.321$, $F = 1.185$, $df = 3$, $N = 21$ per genotype except F413, for which there were 20). The bold line in each box represents the median. Lower and upper box limits indicate the first and third quartiles. The whiskers extend from the top or bottom of the box to the largest or smallest value, but no further than 1.5 times the interquartile range. Outliers beyond the whiskers are indicated by individual points.

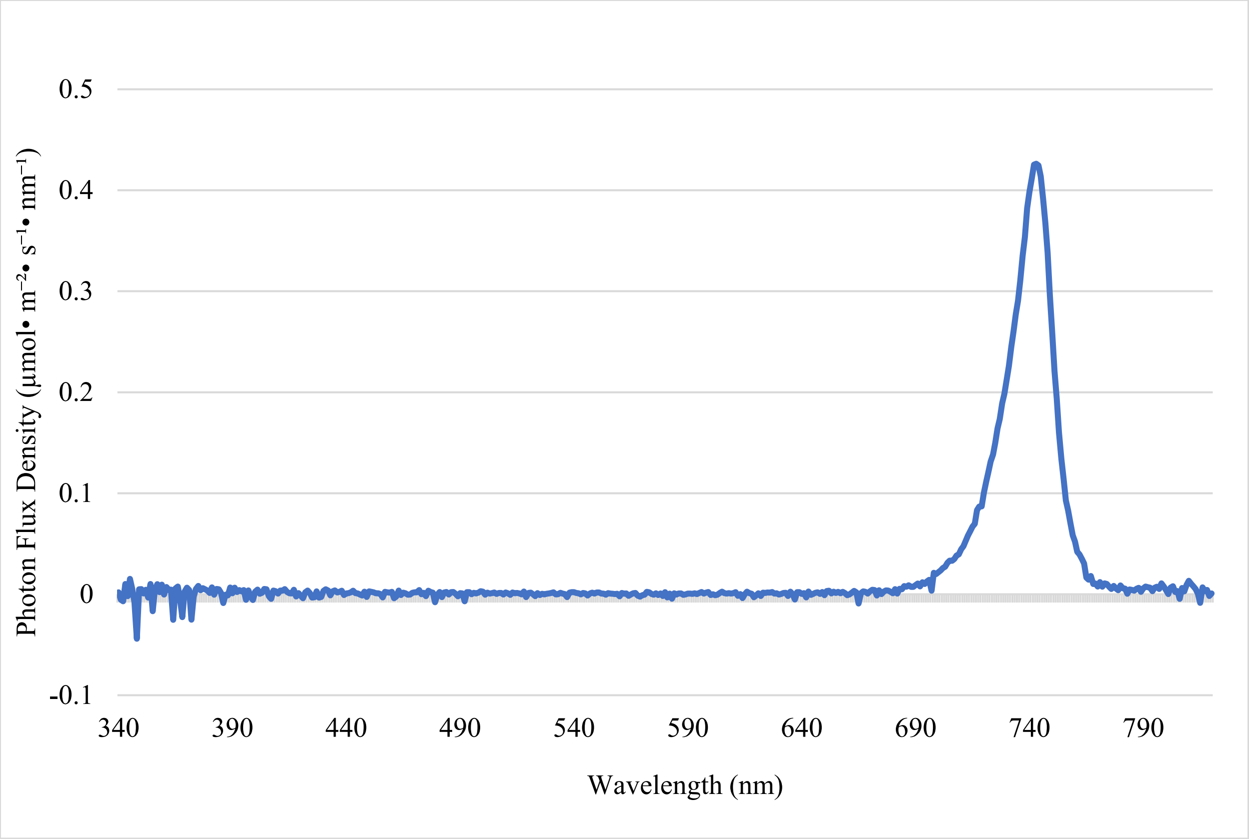

Supplemental Figure S6: Spectral profile of far red light source used for all experiments involving far red light. Spectral profile of the light sources used were measured using a spectroradiometer (SS-110, Apogee Instruments, apogeeinstruments.com) as described in the Methods.

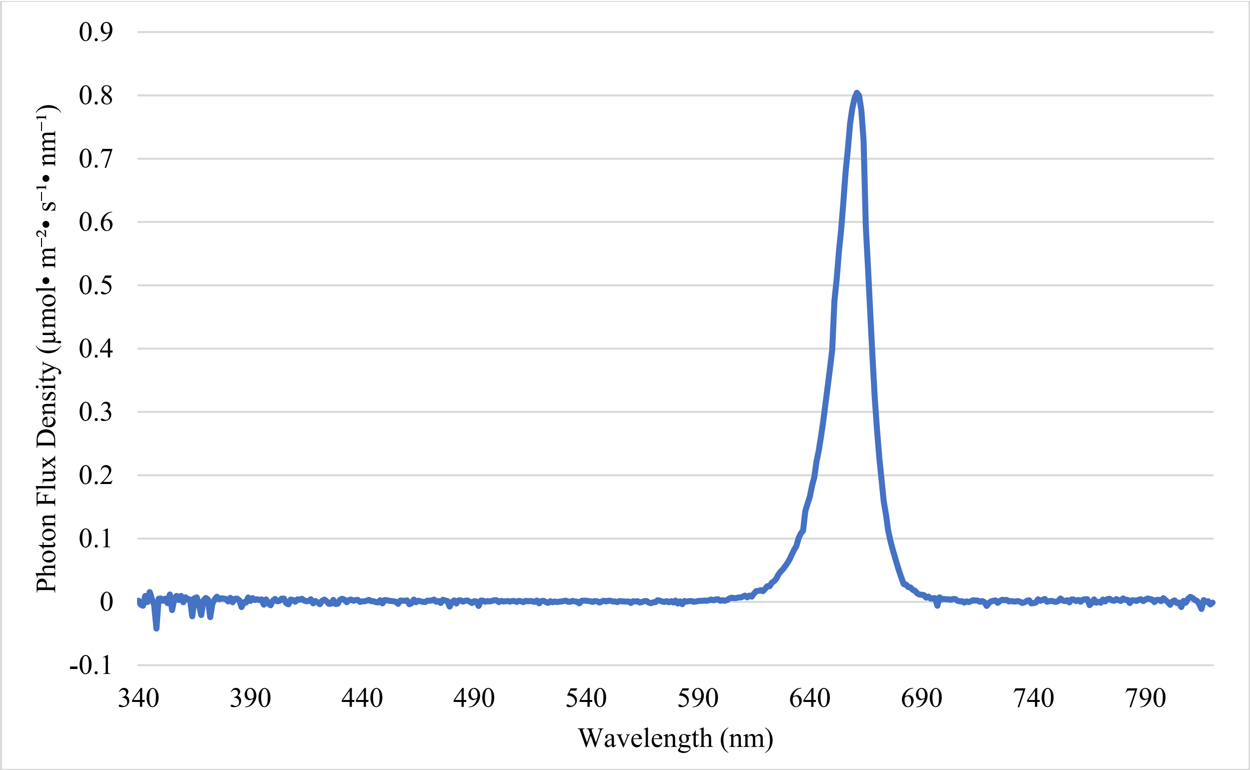

Supplemental Figure S7: Spectral profile of red light source used for all experiments shown in Figures 2, 4, 5, and 6. Spectral profile of the light sources used were measured using a spectroradiometer (SS-110, Apogee Instruments, apogeeinstruments.com) as described in the Methods.

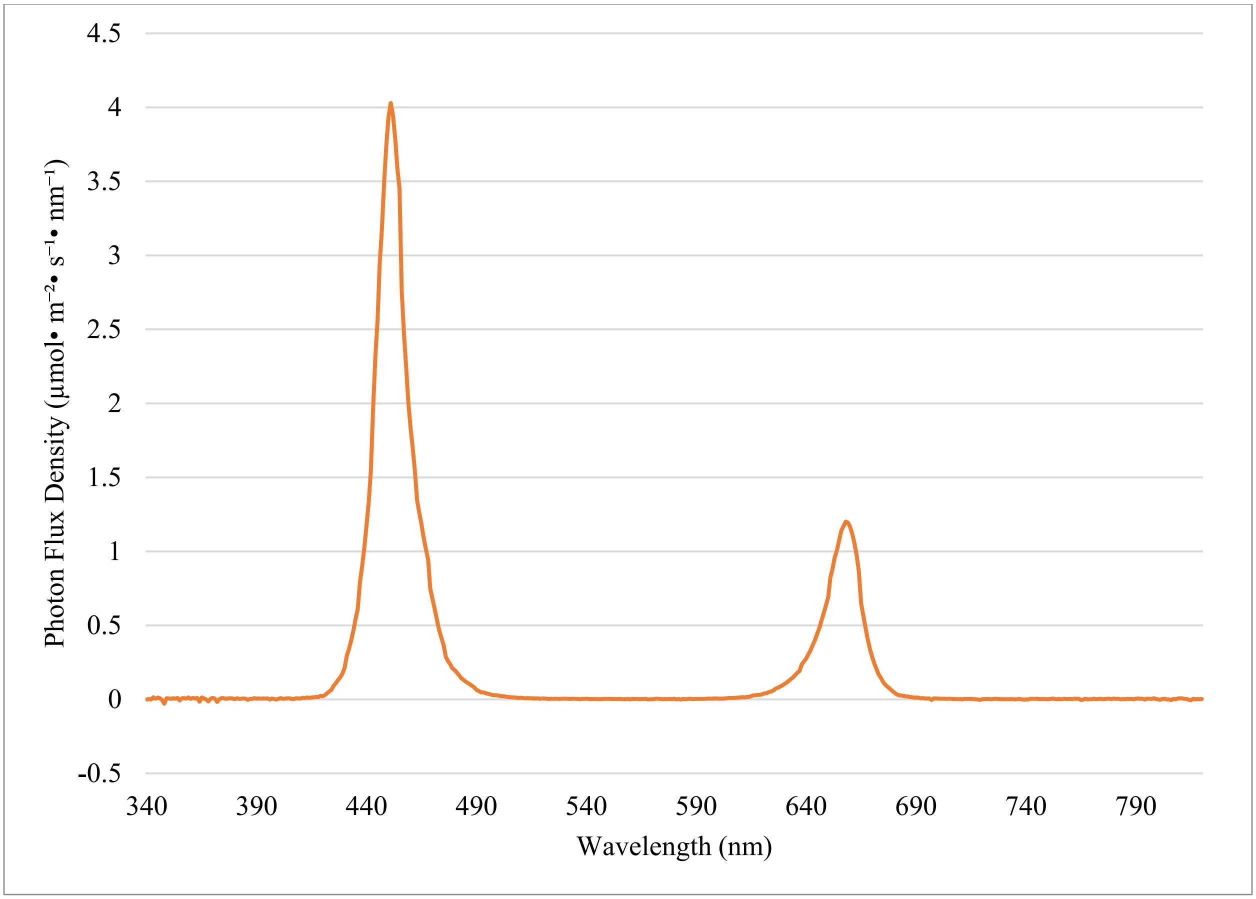

Supplemental Figure S8: Spectral profile of red and blue light sources used in blue/red experiments shown in Supplemental Figure 5 . Spectral profile of the light sources used were measured using a spectroradiometer (SS-110, Apogee Instruments, apogeeinstruments.com) as described in the Methods.

Supplemental Table S1. PCR primers for constructing and genotyping CRISPR-Cas9 mutated lines.

Target	Purpose	Sequence (5' to 3')	Direction
35S promoter and *CAS9* gene	Confirm presence/absence of T-DNA	CATCTCCACTGACGTAAGGG	F
		GTTCTTCTTTATGCTGTGGCG	R
PHYF CRISPR targeted site	Identifying targeted *PHYF* mutation by PCR and sequencing	GATAGAGAGAGATGTCTTCTAGTTC	F
		CTCTTGCTGTTCGATGCTTG	R
PHYF CRISPR targeted site	Construction of gRNA 1 backbone	TGT GGT CTC AAT TGA AGC TTC ATG TGG AGT TTG GTT TTA GAG CTA GAA ATA GCA AG	N/A
PHYF CRISPR targeted site	Construction of gRNA 2 backbone	TGT GGT CTC AAT TGG AAG TCT AAT TCA GCC GTT GTT TTA GAG CTA GAA ATA GCA AG	N/A
